# Supplementary material for: Resting Ca2+ fluxes protect cells from fast mitochondrial fragmentation, cell stress responses, and immediate transcriptional reprogramming
Source: Cell Mol Life Sci. 2025 Jun 14;82(1):238. doi: 10.1007/s00018-025-05745-2 (PMC12167414; doi:10.1007/s00018-025-05745-2)
Supplement: Supplementary file 1 — Supplementary Material 1 [file 18_2025_5745_MOESM1_ESM.docx]

**Supplementary Information**

**Resting Ca^2+^ fluxes protect cells from fast mitochondrial fragmentation, cell stress responses, and immediate transcriptional reprogramming**

Fecher, Sodmann et al. (2025)


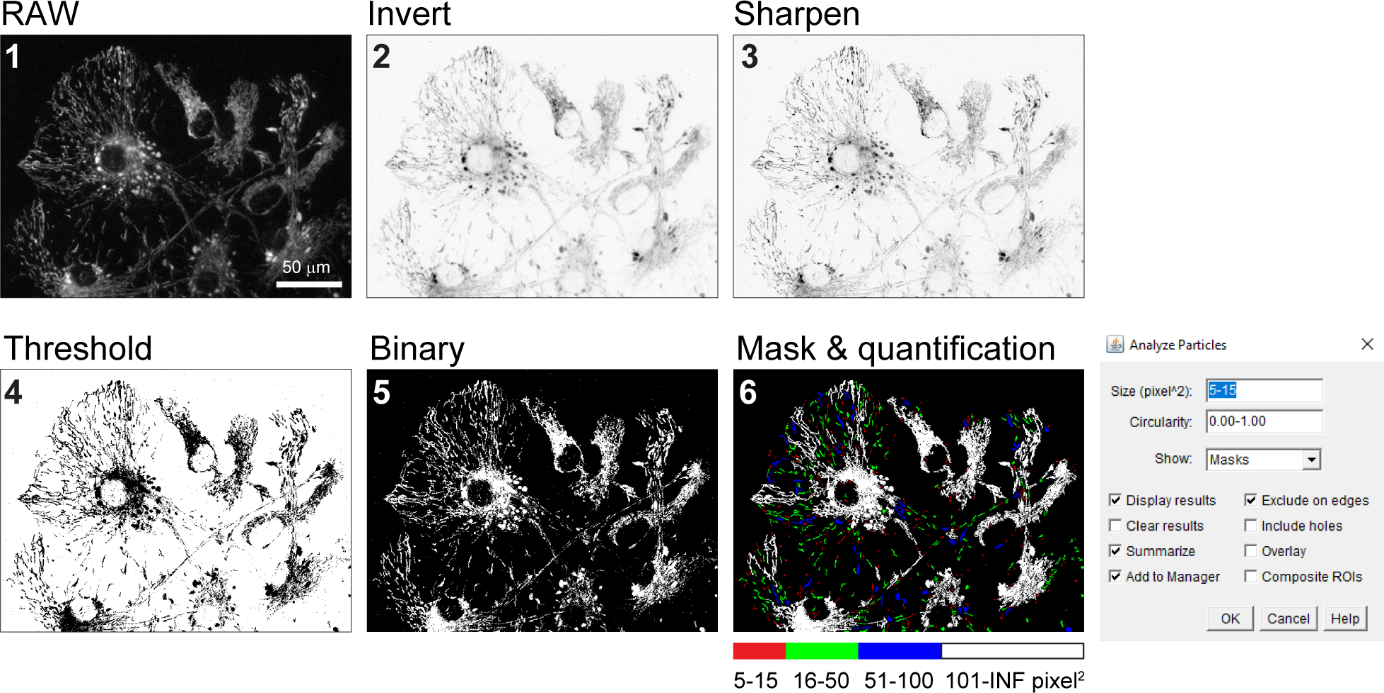


**Figure S1. Quantification of mitochondrial fragmentation.**

Image raw data (8-bit, 1) were inverted and sharpened (2, 3). Signal thresholds (4) were determined to compute binary masks (5) for quantification of mitochondrial particles by size category (6).


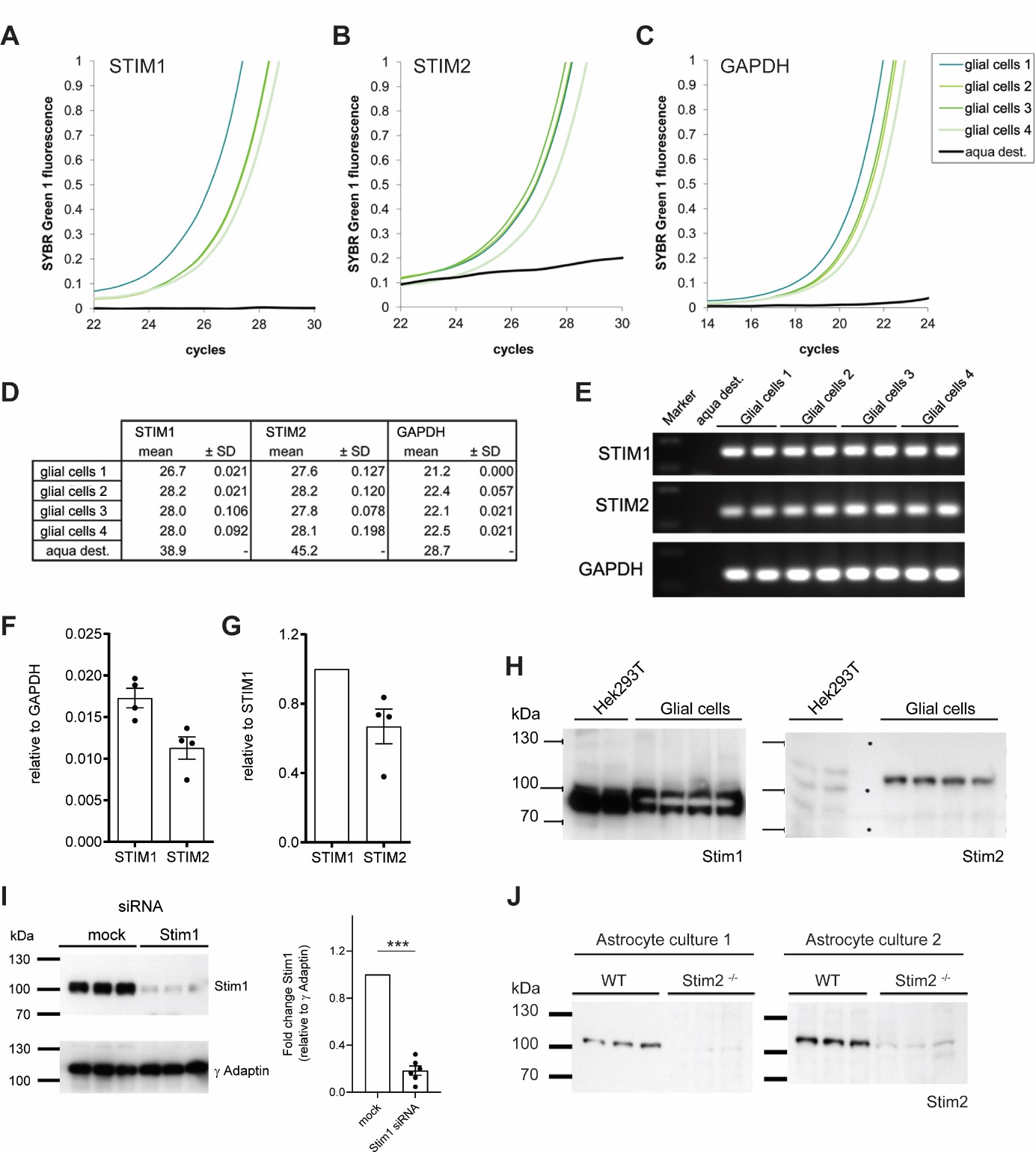


**Figure S2. STIM1 and STIM2 abundance in glial cells, and confirmation of their knock-down and knock-out in culture.**

**A-C.** SYBR green fluorescence traces from qPCR amplification of STIM1 (A), STIM2 (B), and GAPDH (C) in four glial RNA samples from independent cultures. **D**. Quantification of CT values, **E**. visualization of PCR products, and **F, G**. relative quantification of mRNA abundance relative to GAPDH (F) and STIM1 (G). n= 4 biological replicates, Mean ± SEM. **H**. Western blots probing Stim1 and Stim2 proteins in Hek239T cells and primary glial cultures (n= 4 biological replicates). **I**. Western blot and quantification of siRNA mediated knockdown of Stim1 in glial cultures (n= 2 biological replicates measured as technical duplicates). γ Adaptin was used as loading control. Unpaired, two-tailed t-test: t(10) = 20.7, p<0.001, ***. **J**. Western blots from two independent glial cultures generated from Stim2 ^-/-^ pups and Stim2 ^+/+^(WT) littermates.


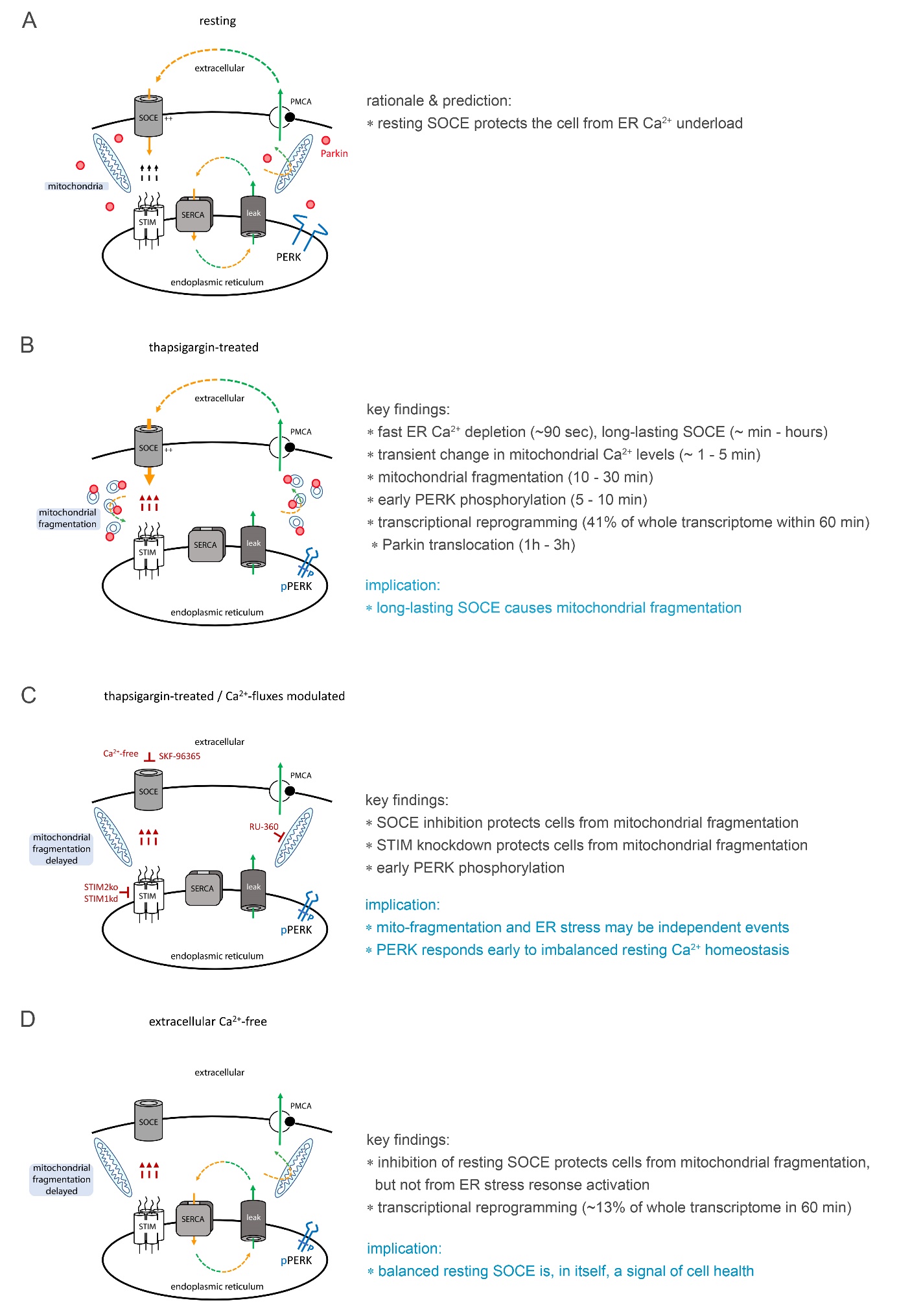


**Figure S3. Minimal models, data summaries, and implications.** **For abbreviations and further details, see Fig. 1A. A.** Resting Ca²⁺ homeostasis in astrocytes is maintained through balanced resting Ca^2+^ fluxes. **B.** Thapsigargin-treatment to block SERCA activity induces multiple biological responses. **C.** SERCA blockade–induced mitochondrial fragmentation occurs within 30 min and can be delayed by inhibiting SOCE, mitochondrial Ca²⁺ uptake, or via STIM protein knockdown. However, these interventions only delay—but do not fully prevent—fragmentation at later time points (> 1h). **D.** Withdrawal of extracellular Ca^2+^ rapidly activates PERK phosphorylation.

*Abbreviations: leak, passive ER Ca^2+^ leak; PERK (=Eif2ak3), pPERK, phosphorylated PERK; PMCA, plasma membrane Ca²⁺ ATPase; RU-360, mitochondrial calcium uptake inhibitor, SERCA, sarcoplasmic/endoplasmic reticulum Ca^2+^-ATPase; SKF-96365; SOCE inhibitor; store-operated Ca^2+^ entry.*
