# Supplementary material for: Resting Ca2+ fluxes protect cells from fast mitochondrial fragmentation, cell stress responses, and immediate transcriptional reprogramming
Source: Cell Mol Life Sci. 2025 Jun 14;82(1):238. doi: 10.1007/s00018-025-05745-2 (PMC12167414; doi:10.1007/s00018-025-05745-2)

Supplementary material:

'Resting Calcium Ion Fluxes Protect Cells from Fast Mitochondrial Fragmentation, Cell Stress Responses, and Immediate Transcriptional Reprogramming'

Fecher, Sodmann et al. (2025)

Presentation of unedited and original western blots

Figure 5A

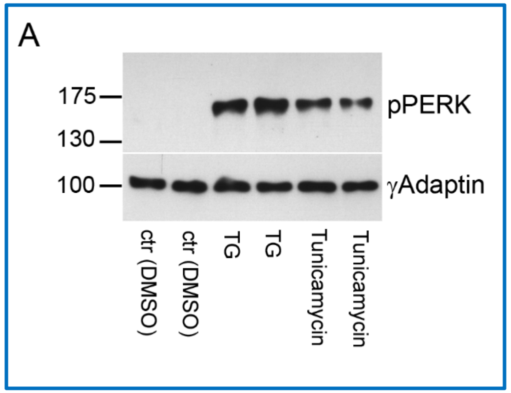

rabbit anti-phospho-PERK (Thr980) (16F8) (1:2,000)
Cell signaling (#3179; RRID: AB_2095853)

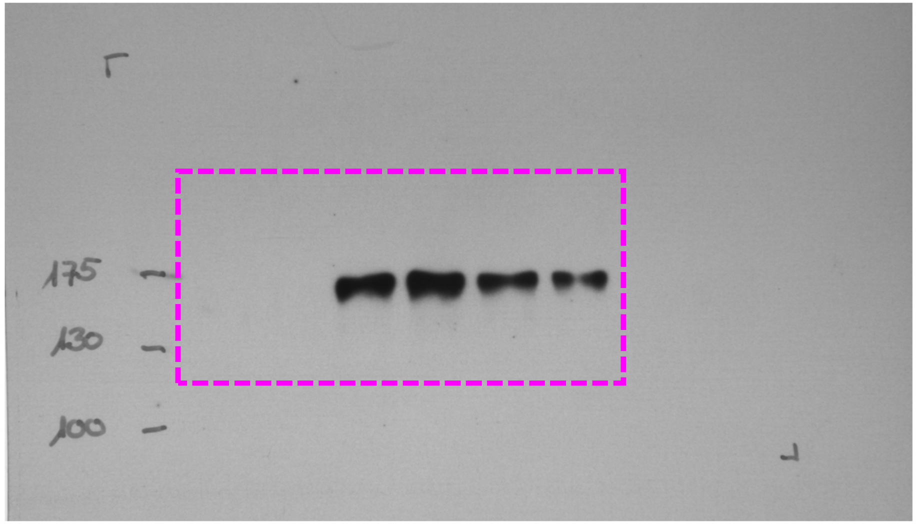

mouse anti-gamma-Adaptin (clone 88) (1:2,000)
BD biosciences (#610385; RRID: AB_397768)

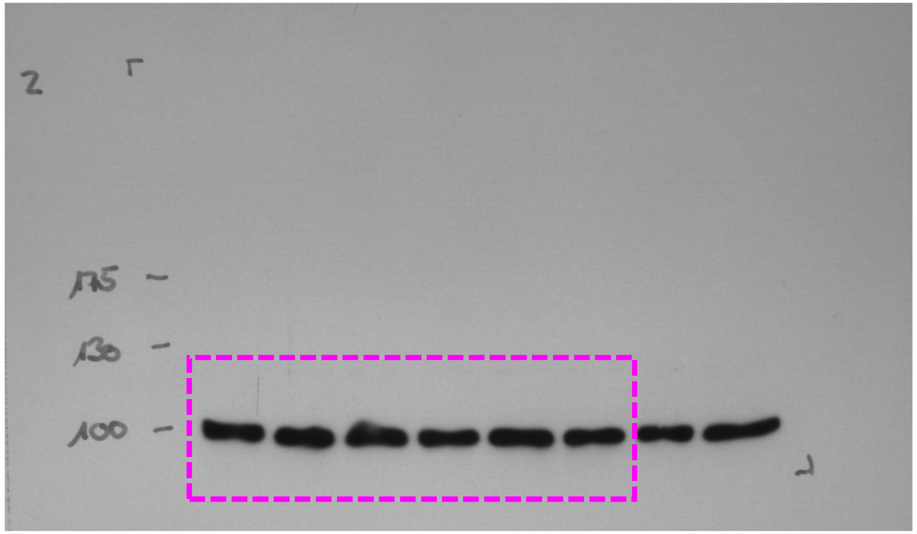

Figure 5B

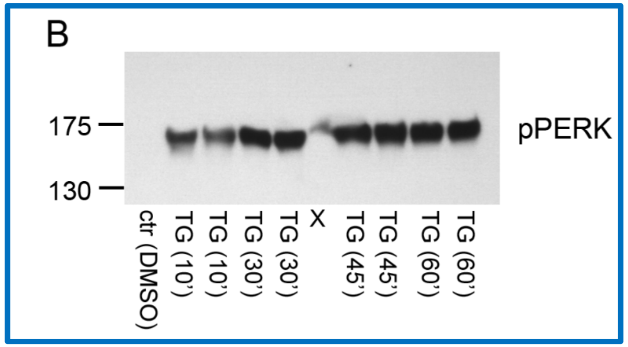

Unedited blots:

rabbit anti-phospho-PERK (Thr980) (16F8) (1:2,000)
Cell signaling (#3179; RRID: AB_2095853)

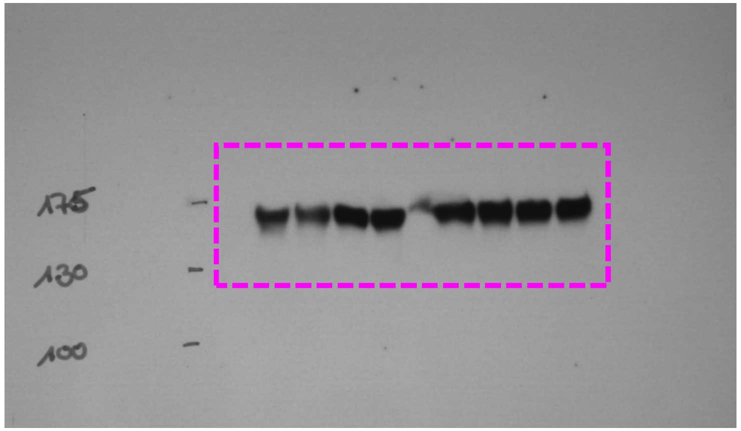

Figure 5C

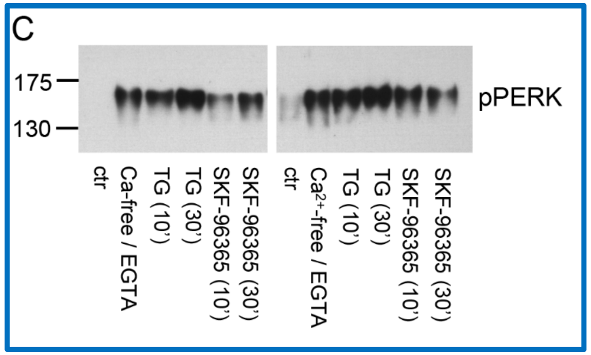

Unedited blots:

rabbit anti-phospho-PERK (Thr980) (16F8) (1:2,000)
Cell signaling (#3179; RRID: AB_2095853)

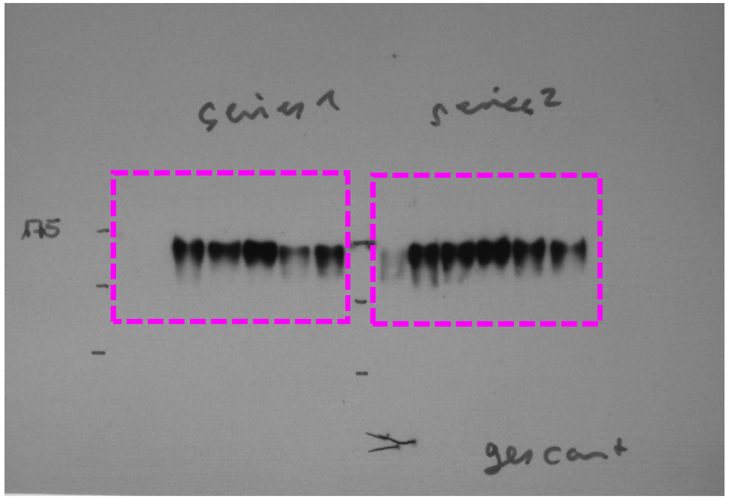

rabbit anti-PERK (C-terminal) (1:2,000)
Sigma-Aldrich (#P0074; RRID: AB_1841092)

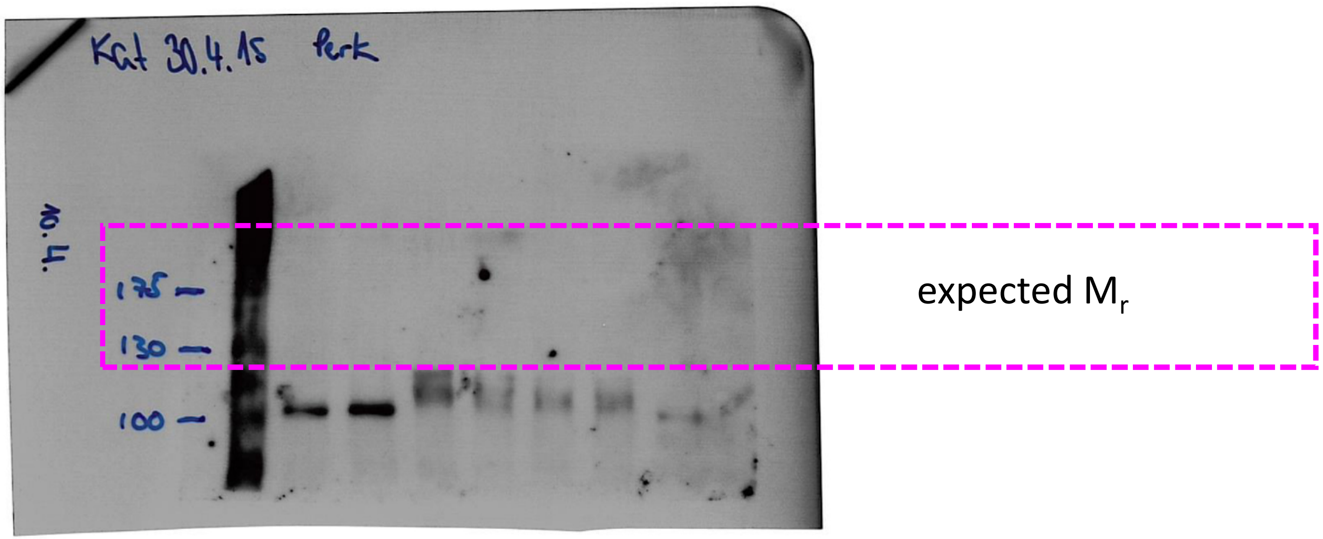

Supplement: Supplementary file 2 — Supplementary Material 2 [file 18_2025_5745_MOESM2_ESM.pdf]
